# Skin-Reducing Mastectomy and Submuscular Expander: A Preliminary Study on Patient-Reported Outcomes

**DOI:** 10.7759/cureus.112892

**Published:** 2026-07-17

**Authors:** Arianna Gatto, Lorenzo Mosiello, Gabriele Negri Zandi, Juste Kaciulyte, Pietro Pecoraro, Leonardo Brambilla, Riccardo Giovanni Isidoro Giovanazzi, Giorgio Pajardi, Andrea Marchesi

**Affiliations:** 1 Plastic and Reconstructive Surgery, Fondazione Istituto di Ricovero e Cura a Carattere Scientifico (IRCCS) San Gerardo dei Tintori, Monza, ITA; 2 Plastic and Reconstructive Surgery, University of Milan, Milan, ITA; 3 Breast Surgery, Azienda Ospedaliera Universitaria Senese, Siena, ITA; 4 Plastic and Reconstructive Surgery, University of Milano-Bicocca, Milan, ITA; 5 Breast Surgery, Fondazione Istituto di Ricovero e Cura a Carattere Scientifico (IRCCS) San Gerardo dei Tintori, Monza, ITA; 6 Hand Surgery and Rehabilitation, Ospedale San Giuseppe, Milano, ITA

**Keywords:** breast plastic surgery, breast-q outcomes, implant breast reconstruction, skin-reducing mastectomy, tissue expander

## Abstract

Background: Immediate implant-based reconstruction in large and ptotic breasts represents a significant surgical challenge. The skin-reducing mastectomy (SRM) pattern has been widely described to manage skin redundancy; however, immediate single-stage prepectoral reconstruction can be burdened by high complication rates in this specific population. While two-stage reconstruction with tissue expanders represents a classic alternative, patient-reported outcomes after SRM using this approach have not been reported in the literature. Materials and methods: We retrospectively reviewed a consecutive cohort of patients with large and ptotic breasts who underwent SRM and two-stage submuscular tissue expander reconstruction between 2022 and 2024. The primary objective was to evaluate postoperative complication rates. Secondary objectives included a preliminary evaluation of patient-reported outcomes six months after definitive implant placement using the validated BREAST-Q Reconstruction Module, alongside a narrative benchmarking of our results against historical literature series. Tissue expanders were placed in a dual-plane submuscular-subdermal pocket without the use of acellular dermal matrices. Results: A total of 22 patients were included. Twenty patients (90.9%) successfully completed the two-stage reconstruction, while two (9.1%) experienced expander failure requiring explantation and autologous salvage, thereby excluding them from the BREAST-Q analysis. The total complication rate was 27.3% (6/22), and the surgical revision/implant failure rate was 9.1% (2/22). The BREAST-Q analysis highlighted high satisfaction scores regarding implants and postoperative breast appearance while revealing lower scores related to sexual well-being and cancer concerns. The literature review identified four relevant historical series characterized by small cohorts and the complete absence of patient-reported outcome data.

Conclusions: The use of a two-stage submuscular tissue expander after SRM in patients with ptotic, large breasts represents a feasible and reliable alternative for managing skin redundancy with an acceptable complication profile in a high-risk population. Given the retrospective and uncontrolled nature of this preliminary study, further prospective comparative trials are required to establish definitive comparative effectiveness.

## Introduction

A variety of reconstructive techniques have been described for breast reconstruction after mastectomy. These range from direct implant-based reconstruction, performed in either one or two stages, to more complex procedures involving autologous tissue transfer, either as pedicled or free flaps. Hybrid approaches combining prosthetic and autologous reconstruction may also be considered, depending on patient-specific factors and reconstructive objectives [1]. New oncoplastic principles have also been applied after total mastectomy, as seen in the Goldilocks mastectomy, which consists of remodeling the residual skin and subcutaneous tissue in patients with large and ptotic breasts [2].

Tumor size and location, breast volume, and the anticipated need for neoadjuvant or adjuvant therapies are the main factors influencing surgical decision-making. In prosthetic reconstruction of medium to large breasts with varying degrees of ptosis, an associated skin-reduction procedure is often required to achieve an optimal aesthetic outcome. The most frequently used approaches include a vertical pattern or an inverted-T pattern, often combined with contralateral symmetrization to improve overall cosmetic symmetry [3].

Among these techniques, the skin-reducing mastectomy (SRM) pattern has been widely described [4-6], but in large and ptotic breasts, an immediate single-stage implant-based reconstruction remains highly challenging. Particular concern has been raised regarding the relatively high incidence of skin flap necrosis, especially at the T-junction, when performing SRM with immediate single-stage silicone implant reconstruction due to excessive skin tension at the lower pole [7]. For this reason, a two-stage reconstruction combining a SRM pattern with temporary tissue expander placement has been proposed as a safer alternative [8].

In large and ptotic breasts, a Wise-pattern SRM followed by two-stage reconstruction allows the surgeon to reduce the vertical and horizontal dimensions of the skin flaps, thereby improving their vascular supply, particularly at the T-scar. Concurrently, the gradual intraoperative and postoperative filling of a tissue expander helps reduce immediate mechanical tension on the lower-pole skin flaps, mitigating the acute risk of wound dehiscence and subsequent implant exposure.

The primary objective of this study was to retrospectively evaluate the clinical outcomes and complication rates of a consecutive cohort of patients with large and ptotic breasts who underwent SRM and two-stage reconstruction with a submuscular tissue expander at our institution. The secondary objectives were (1) to report preliminary postoperative patient-reported outcomes using the validated BREAST-Q Reconstruction Module and (2) to perform a literature review to contextualize our clinical findings against historical benchmarks. To the best of our knowledge, this is the first study to incorporate patient-reported outcome data via the BREAST-Q questionnaire specifically for SRM followed by a two-stage reconstruction without the use of acellular dermal matrices (ADM).

## Materials and methods

Study design and patient selection

This retrospective cohort study included all consecutive patients who underwent SRM followed by two-stage reconstruction with tissue expanders at the IRCCS Foundation S. Gerardo Dei Tintori (Monza, Italy) between 2022 and 2024. All participants provided written informed consent for both the surgical procedures and the pseudonymized use of pre- and postoperative clinical data for scientific purposes. The study protocol was conducted in strict accordance with the ethical tenets of the Declaration of Helsinki.

Inclusion and exclusion criteria

Patients were eligible for inclusion if they presented with unilateral breast cancer (stages Tis to T3) and co-existing significant breast ptosis. Candidates for SRM were formally defined as those with a nipple-to-inframammary fold (IMF) distance ≥8 cm and a sternal notch-to-nipple distance ≥25 cm. Cases were excluded from this surgical protocol if there was oncological skin or subcutaneous involvement beyond the retroareolar area that necessitated wide skin resections compromising the standard Wise-pattern skin reduction.

Surgical technique and perioperative protocol

**Figure 1:**
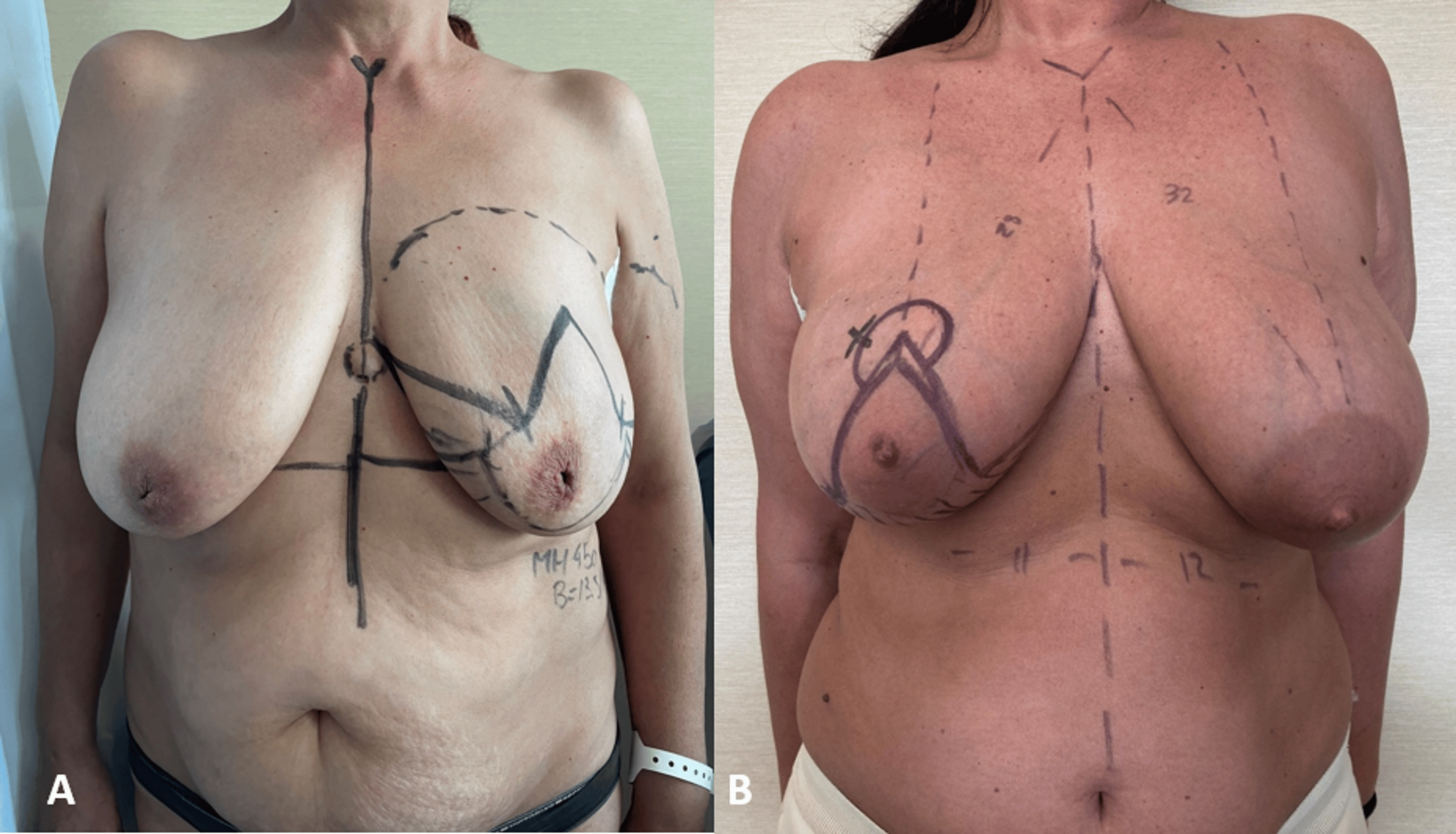
Preoperative planning of SRM in Patient 8 (A) and Patient 11 (B) SRM: skin-reducing mastectomy

With the patient standing facing the surgeon, the midclavicular line was marked first. The Pitanguy maneuver was performed to identify the new nipple-areola complex (NAC) position, typically located 19-23 cm along this line. Vertical lines (5-6 cm) were drawn downward from the new NAC center, and horizontal lines connected these caudal points to the IMF, following a standard Wise-pattern design. In all cases included in this series, the native NAC was excised for oncological safety. The area between the vertical lines and the IMF was delineated for de-epithelialization, forming a vascularized inferior dermal flap to be incorporated into the dermomuscular pouch (Figure 1).

Following total mastectomy, a retromuscular pocket was created posterior to the pectoralis major muscle. The muscle was gently detached from its caudal IMF origin, and its inferomedial sternal fibers were partially released to minimize animation deformity post-implantation. After positioning the tissue expander, the inferior dermal flap was securely sutured to the lower free margin of the pectoralis major muscle, ensuring complete coverage of the device with well-vascularized tissue. The overlying skin flaps were then closed in a standard inverted-T pattern. Two-stage reconstruction with temporary expanders was systematically selected over direct-to-implant procedures due to severe breast ptosis and significant skin redundancy requiring a Wise pattern, where immediate placement of a permanent prosthesis would have induced excessive lower-pole tension or compromised skin flap perfusion.

All patients received uniform perioperative intravenous antibiotic prophylaxis at the time of anesthesia induction and for 24 hours postoperatively. Closed-suction drains were placed routinely in both the retromuscular space and the subcutaneous plane; drains were removed postoperatively once the 24-hour output fell below 30 cc for two consecutive days.

Clinical outcome measures

Patient medical records were reviewed for demographics, follow-up duration, and surgical complications. Complications were strictly classified as minor (managed conservatively in an outpatient setting without surgical intervention) or major (requiring return to the operating room for surgical revision, debridement, or implant explantation). Reconstructive failure was defined as the unexpected removal of a tissue expander without replacement.

Patient-reported outcomes (BREAST-Q)

Patient satisfaction and health-related quality of life were evaluated using the official, licensed BREAST-Q (postoperative version). The officially validated Italian translation was administered. To minimize interviewer and social desirability bias, the questionnaires were self-completed by patients in a private room during their routine follow-up visit scheduled exactly six months after definitive prosthesis placement.

Raw responses from individual scales were converted into standard 0-100 Q-scores using the official BREAST-Q scoring guidelines and software. In accordance with the psychometric design of the tool, each scale was scored independently, and no global overall BREAST-Q score was computed. Missing items were managed strictly in accordance with the user manual [9].

Statistical analysis

To place our preliminary results in the context of the historical literature, Fisher’s exact test was used to compare complication rates between our series and previously published cohorts. A p-value less than 0.05 was considered statistically significant. All analyses were performed using SAS software version 9.4 (SAS Institute Inc., Cary, NC, USA).

Literature review

The systematic review was conducted in accordance with the Preferred Reporting Items for Systematic Reviews and Meta-Analyses (PRISMA) guidelines. Two authors (A.G. and P.P.) searched PubMed, EMBASE, and the Cochrane electronic database independently to identify eligible articles. The search parameters used were the following: "(skin-reducing mastectomy) OR (skin reduction) AND (tissue expander)." All articles published between 1st January 1986 and 1st May 2024 that met the inclusion criteria were included in the review.

Criteria for inclusion in the review were original articles reporting case series of SRM and two-stage reconstruction with a tissue expander and subsequent prosthesis positioning. Criteria for article exclusion were non-original articles, letters to the editor, case reports, literature reviews, or meta-analyses without a clinical case series, articles in languages other than English, articles not available in full text, single-stage reconstruction, articles that do not distinguish complications from different surgical techniques, and case series reporting reconstruction with a Becker expander.

## Results

Retrospective case series clinical outcomes

From 2022 to 2024, 22 consecutive patients underwent unilateral SRM and two-stage submuscular reconstruction with tissue expanders at our center. The median age of the cohort was 55.6 years, and the median follow-up duration was 35.6 months (range: 12-47 months). The baseline clinical characteristics and overall complication rates are detailed in Table 1.

**Table 1:** Patient characteristics and clinical outcomes of the cohort (N = 22) Data refer to patients who underwent unilateral SRM and two-stage reconstruction with tissue expanders between 2022 and 2024. ¹ The 2 patients who required expander explantation due to complications underwent autologous reconstruction and were excluded from the BREAST-Q analysis. SRM: skin-reducing mastectomy

Characteristic/clinical outcome	Value/n (%)
Demographics and follow-up	
Age (years), median	55.6
Follow-up (months), median (range)	35.6 (12–47)
Reconstruction outcomes	
Completion of second stage	20 (90.9%)
Expander failure with explantation¹	2 (9.1%)
Surgical complications	
Total complication rate	6 (27.3%)
Minor complications	4 (18.2%)
Seroma	3 (13.6%)
Skin flap necrosis	1 (4.5%)
Major complications	2 (9.1%)
Infection	1 (4.5%)
Skin flap necrosis	1 (4.5%)
Surgical revision rate	2 (9.1%)

**Figure 2:**
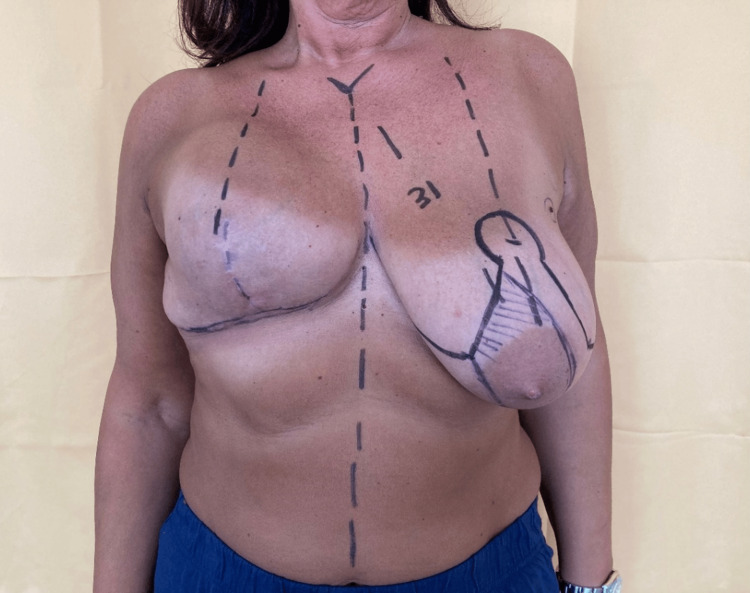
Preoperative planning for second-stage reconstruction after complete expansion of the implant in Patient 11

**Figure 3:**
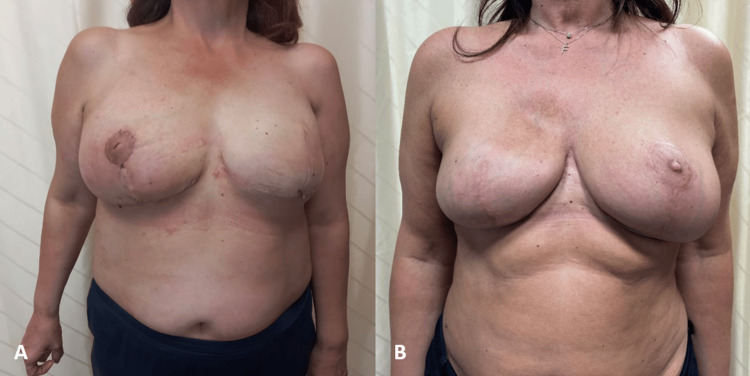
Postoperative images six months after the second procedure in Patient 8 (A) and Patient 11 (B)

Twenty patients (90.9%) successfully completed the second-stage definitive implant exchange (Figure 2). Two patients (9.1%) experienced major complications during the expansion phase, consisting of skin flap necrosis and severe infection leading to expander explantation. Both patients subsequently underwent successful autologous free-flap salvage reconstruction. Because they did not complete the two-stage reconstruction, these two patients were excluded from the BREAST-Q analysis, creating an inherent survivorship bias that must be considered when interpreting the patient-reported metrics. Representative postoperative outcomes six months after the second-stage procedure are shown in Figure 3.

Complications included four minor complications: three seromas (13.6%) and one skin flap necrosis (4.5%); and two major complications: one infection (4.5%) and one skin flap necrosis (4.5%). Total complication rate was 6/22 (27.3%); the surgical revision/implant failure rate was 2/22 (9.1%) (Table 1).

Literature review

A total of 142 articles were selected from the PubMed database, seven articles were selected from the Cochrane database, and 225 articles were selected from the Embase database. Duplicated articles were identified and removed. The remaining articles underwent a first screening through title and abstract review, followed by a second screening through full-text reading by two authors independently (A.G. and P.P.). In the end, 48 duplicated articles were eliminated; 306 articles were not considered, as they did not suit the inclusion criteria, and 20 more articles were not considered after a full-text reading evaluation. At the end of the review, four articles met the inclusion criteria and were selected.

Pairwise comparisons

No significant differences were found versus Payne et al., Losken et al., and Hammond et al. for overall complications, flap necrosis, seroma/hematoma, infection, implant failure, or reintervention (Fisher’s exact test) [8,10,11]. Significant differences favored our series over Komorowska-Timek et al. for overall complications (OR 5.18, p = 0.004), flap necrosis (OR 9.64, p = 0.003), and infections (OR 8.80, p = 0.003) [12]. No differences in seroma/hematoma or implant failure.

Breast-Q results

The BREAST-Q was completed by the 20 patients who successfully achieved definitive implant exchange. The standardized domain scores are summarized in Table 2 using standard core scale nomenclature.

**Table 2:** Patient-reported outcomes using the BREAST-Q (N = 20) Summary of BREAST-Q domain scores for patients who underwent successful prosthetic breast reconstruction (N = 20). Data are reported as mean and absolute range (minimum-maximum). Domains are ordered by descending mean score.

BREAST-Q	Mean score	Range
Satisfaction with Implants	87	42-100
Psychosocial well-being	77	45-100
Satisfaction with breast	75	40-100
Physical well-being: adverse conditions (breast symptoms)	72	42-100
Sexual well-being	63	38-85
Cancer concerns (supplementary module)	53	35-83

The highest mean satisfaction scores were reported for the satisfaction with implants scale (87; range: 42-100). The psychosocial well-being scale yielded a mean score of 77 (range: 45-100), closely followed by satisfaction with breasts (75; range: 40-100). Physical tolerance was acceptable, with a mean score of 72 (range: 42-100) on the physical well-being: adverse conditions (breast symptoms) scale. Conversely, the lowest scores within our population were observed for sexual well-being (63; range: 38-85) and the supplementary cancer concerns scale (53; range: 35-83), identifying key areas where targeted psychosocial support may be required (Table 2).

## Discussion

Plastic surgeons performing immediate breast reconstruction must balance several concurrent goals: minimizing postoperative complications, restoring the physical contour of the breast, and preserving long-term patient-reported quality of life. While many centers currently consider single-stage prepectoral direct-to-implant reconstruction to be a highly effective approach [13], a specific subpopulation of patients presenting with voluminous and ptotic breasts remains highly vulnerable to ischemic complications. When a Wise-pattern skin reduction is performed, these individuals are particularly prone to skin flap necrosis at the T-junction, as the large flap dimensions can compromise peripheral perfusion under increased tension.

In this challenging population, a two-stage approach using a temporary tissue expander after SRM offers important mechanical and ischemic advantages. Staging the reconstruction facilitates the gradual expansion and adaptation of the reduced mastectomy flaps, significantly reduces immediate postoperative skin tension at the lower pole, provides a protected retromuscular housing for the expander, and minimizes the risk of major wound dehiscence or early prosthetic exposure. Furthermore, high-quality comparative data combining SRM with immediate single-stage prepectoral reconstruction are currently lacking in the literature; the few small series available report total complication rates ranging from 16% to 25% [14,15] without long-term tracking, making definitive clinical comparisons difficult.

Our review of the literature highlighted that very few studies have specifically examined the combination of a Wise-pattern SRM and a two-stage reconstruction, and existing publications are limited by modest sample sizes. Crucially, none of the previously published series incorporated validated patient-reported outcome measures, leaving a significant gap in understanding of subjective patient satisfaction.

A comparison of the historical series reveals a wide range of clinical outcomes (Table 3). Payne et al. [8] reported on 47 breasts, observing 16 minor and four major complications, representing an overall complication rate of 42.5% and a reintervention rate of 8.5%. Losken et al. [10] evaluated 34 breasts, reporting 10 minor and six major complications (47% total complication rate) with a 17.6% reintervention rate. Hammond et al. [11] reviewed a small series of 10 breasts, reporting a 20% overall complication rate (including one major flap necrosis and one expander explantation) and a 20% reintervention rate. Finally, Komorowska-Timek et al. [12] reported on 45 breasts, demonstrating a high incidence of adverse events, including a 40% rate of flap necrosis, a 37.8% rate of infections, and a 17.8% expander removal rate, culminating in an overall complication rate of 62.2%. These findings are summarized in Table 3.

**Table 3:** Comparison of the current study with selected studies from the literature review

	N° of operated breasts	Flap necrosis	Seroma/hematoma	Infection	Implant failure	Minor complications (total)	Major complications (total)	Total number of complications	Reintervention rate
Payne et al., 2022 [8]	47	15 (31.9%)	1 (2.1%)	2 (4.2%)	2 (4.2%)	16 (34%) (15 flap necrosis + 1 seroma/hematoma)	4 (8.5%) (2 infections + 2 implant failure)	20 (42.5%)	4 (8.5%)
Losken et al., 2010 [10]	34	5 (14.7%)	6 (17.65%)	4 (11.75%)	1 (2.94%)	10 (29.41%)	6 (17.65%) (3 infections + 2 skin flap necrosis + 1 skin flap necrosis leading to infection)	16 (47%)	6 (17.65%)
Hammond et al., 2002 [11]	10	1 (10%)	-	-	1 (10%)	0	2 (20%) (1 flap necrosis + 1 implant failure/removal)	2 (20%)	2 (20%)
Komorowska-Timek et al., 2019 [12]	45	18 (40%)	2 (4.4%)	17 (37.8%)	8 (17.8%)	Not specified	Not specified	28 (62.2%) (reported as “any complication”)	Not specified
Current study	22	2 (9.1%)	3 (13.6%)	1 (4.5%)	2 (9.1%)	4 (18.2%) (3 seromas + 1 skin flap necrosis)	2 (9.1%) (1 infection + 1 skin flap necrosis)	6 (27.3%)	2 (9.1%)

Our observed total complication rate of 27.3% (6/22) is clinically acceptable and falls within the lower end of the ranges reported in the historical literature. Among the six complications observed in our series, the two cases of full-thickness skin flap necrosis and deep infection that led to temporary reconstructive failure (9.1%) were successfully managed via delayed autologous free-flap transfer. This indicates that while the technique is reliable for remodeling severe skin redundancy, it requires careful patient selection and a conservative post-mastectomy expansion protocol.

Regarding our patient-reported metrics, successfully reconstructed patients demonstrated encouraging satisfaction scores, particularly concerning breast contour and implant aesthetics. However, the interpretation of our BREAST-Q data must be approached with caution due to several significant methodological limitations. First, our study is an uncontrolled, retrospective case series with a modest cohort size (N = 22), reflecting the highly selective nature of this procedure at our institution. Second, the lack of baseline, preoperative BREAST-Q scores introduces potential recall bias regarding patients' retrospective perceptions of their pre-surgical breasts.

Importantly, our survey data is subject to a distinct survivorship bias. Because the BREAST-Q was administered exclusively at the six-month postoperative mark to patients who successfully completed the implant exchange (n = 20), the negative physical and psychological impacts experienced by the two patients who suffered early expander loss are not captured in the mean scores. This omission likely inflates the reported satisfaction rates. Finally, our study lacked a contemporaneous, active control group, did not evaluate specific individual risk factors due to the small sample size, and did not include an objective, independent panel to blind-rate the aesthetic outcomes.

To address these limitations and eliminate recall and survivorship biases, an institutional prospective study is currently underway. This ongoing trial features a larger sample size. It incorporates mandatory preoperative baseline BREAST-Q testing and longitudinal postoperative follow-ups for all enrolled patients, regardless of their final reconstructive track. This will help clarify the role of two-stage submuscular SRM reconstruction within the contemporary oncoplastic landscape.

## Conclusions

According to our preliminary findings and historical literature benchmarking, combining a Wise-pattern SRM with a two-stage submuscular tissue expander reconstruction appears to be a feasible and effective alternative for patients presenting with large, ptotic breasts who are poor candidates for direct-to-implant protocols. The observed complication rate is acceptable given the high-risk nature of this population, and patients report high satisfaction with their final breast shape and physical well-being. However, given the retrospective design and clear survivorship bias of this small cohort, these results must be interpreted as hypothesis-generating. Larger, prospective controlled trials remain necessary to confirm long-term comparative efficacy and safety.
